# Editorial: Indigenous aging

**DOI:** 10.3389/fpubh.2022.1111672

**Published:** 2022-12-20

**Authors:** R. Turner Goins, Louise C. Parr-Brownlie, Kylie Radford

**Affiliations:** ^1^Department of Social Work, College of Health and Human Sciences, Western Carolina University, Cullowhee, NC, United States; ^2^Department of Anatomy, Brain Health Research Centre, School of Biomedical Sciences, University of Otago, Dunedin, New Zealand; ^3^Ageing Well National Science Challenge, University of Otago, Dunedin, New Zealand; ^4^Neuroscience Research Australia, Sydney, NSW, Australia; ^5^Ageing Futures Institute, University of New South Wales, Sydney, NSW, Australia

**Keywords:** gerontology and geriatrics, indigenous peoples, dementia, health, care access, social support, global health equity

The global number of older persons is projected to more than double over the next 30 years, reaching over 1.5 billion persons by 2050 (1). Indigenous Peoples are distinct social and cultural groups that share collective ancestral ties to the lands and natural resources where they live, occupy, or from which they have been displaced. Their health and wellbeing are inextricably linked to their cultures. Worldwide, the colonization and marginalization of Indigenous Peoples have contributed to poorer health, higher rates of disability and reduced quality of life, and increased likelihood to die at younger ages than their non-indigenous counterparts (2–4). The health profile of older Indigenous Persons coupled with their expected rapid population growth, warrants efforts to better understand the Indigenous aging experience to meet their growing public health needs and ensure older Indigenous Peoples' right to equitable healthy aging. Yet, there is a relatively limited amount of research worldwide that has focused on health-related concerns of older Indigenous Peoples, including health and care access inequities.

The Research Topic “*Indigenous aging*” included eight manuscripts reporting on research findings regarding older Indigenous peoples with three overarching themes, including (1) health and health determinants, (2) healthcare access, and (3) the importance of solutions building upon inherent strengths. The foci of these manuscripts were diverse, broadly including:

- Indigenous knowledge of healthy aging to inform the design of what is important (Quigley et al.).
- Health-related needs and care access (Goins et al.; Jaramillo et al.; Kawakami et al.; Lewis et al.).
- Education of older Indigenous peoples (Oetzel et al.) and of community health workers and carers for Indigenous peoples living with dementia (Goldberg et al.).
- Protective factors for dementia and cognitive decline (Thompson et al.).

## Health and health determinants

The burden of colonization, disconnection from lands, and degradation of language, culture and values has, and continues to negatively impact Indigenous health. Increased appreciation and understanding of facilitators of healthy Indigenous aging can help direct culturally appropriate and effective healthcare (Quigley et al.). A holistic perspective of aging, at individual and community levels, that acknowledges the importance of physical, mental, spritual health and social connections (Goins et al.; Kawakami et al.; Quigley et al.) is critical. Older Indigenous persons experience disparities in well-established social determinants of health and wellbeing such as education and physical activity (Jaramillo et al.; Thompson et al.), but also have unique social determinants of health and wellbeing such as community interdependence and culture (Goins et al.; Oetzel et al.; Quigley et al.). To continue to improve our understandings of health and determinants of health, measurements of these determinants need to be appropriate, and may need to be specifically developed for diverse Indigenous populations (Lewis et al.; Oetzel et al.). Moreover, interventional work designed to improve health and wellbeing among aging Indigenous persons should be tailored for and delivered by Indigenous peoples and/or providers (Lewis et al.).

## Healthcare access

Given the health inquities experienced by older Indigenous peoples, it is important that formal healthcare services are culturally safe and centered to maximize health and wellbeing (Goins et al.; Goldberg et al.; Quigley et al.). Indigenous peoples need regular, consistent healthcare from providers who get to know their patients and seek to understand the culture, protocols, and traditional medicines so as to build a trusting relationship (Jaramillo et al.; Kawakami et al.). For many older Indigenous adults, the transactional nature of Western healthcare is ineffective (Goins et al.; Quigley et al.) and compassionate healthcare professionals are preferred (Kawakami et al.). Often, Indigenous communities are served by transient Western doctors with limited access to specialists, which negatively affects cultivating trusting patient-provider relationships (Jaramillo et al.; Kawakami et al.). Ideally, Indigenous health practitioners, cognisant of their culture, language and protocols, are the healthcare provider and advocate for the wellbeing of Indigenous older adults.

## Solutions building upon inherent strengths

Reciprocity and interconnectedness are cultural values prevalent in Indigenous communities, which support strong and trusting relationships. Social support in particular is a strength common across Indigenous cultures that deserves greater attention in seeking innovative approaches to improving health and wellbeing (Goins et al.; Oetzel et al.). Alongside this, the knowledge gained in healthcare and research settings needs to be shared in traditional ways, such as a storytelling format (Kawakami et al.). The information about an Indigenous older person's health may be woven into a creation story or shared in metaphorical ways that resonate with the person. A corollary of this is that researchers working with and for Indigenous persons need to share information with them in traditional formats (e.g., face to face meetings) so that they, and their family, can make informed decisions about healthcare. Such approaches maintain and strengthen foundational relationships and community bonds. Finally, Indigenous healthcare professionals and researchers will need to share their research findings in academic journals, and in reports that can drive changes in health policy, delivery, and care.

Collectively, these manuscripts provide evidence to fill gaps in healthcare delivery for Indigenous peoples, and provide solutions to produce equitable health and wellbeing outcomes, which may need to be refined according to sociocultural context, in partnership with older Indigenous peoples and their communities. In summary, more work is needed to identify existing assets and strengths in Indigenous communities that can be leveraged to support better health-related outcomes. Greater understandings on these fronts can be used to inform the development and implementation of successful health interventions for this population and are important steps to be taken to fully and equitably meet the health-related needs of older people, as required by the United Nations Declaration on the Rights of Indigenous peoples (5).

## Author contributions

All authors listed have made a substantial, direct, and intellectual contribution to the work and approved it for publication.
